# I Don’t Care Who You Are: Adult Respondent Selection Does Not Alter Child Deprivation Estimates

**DOI:** 10.1007/s12187-023-10025-1

**Published:** 2023-03-20

**Authors:** Alba Lanau, Mitieli Cama, Dave Gordon

**Affiliations:** 1 Universitat Pompeu Fabra, Barcelona, Spain; 2 Fiji Bureau of Statistics (Former Chief Statistician – Household Survey Division), Suva, Fiji; 3 University of Bristol, Bristol, UK

**Keywords:** Child poverty, Deprivation, Respondent selection, Mother

## Abstract

The last decade has seen strong developments in child poverty measurement. Children are largely recognised to have age-specific needs, which has led to the development of child-specific poverty measures. One of the current ongoing debates is how best to collect that information. Questions regarding child deprivation are most often answered by a reference adult and assume that adult respondents provide accurate reports about their children’s needs. These assumptions have largely gone untested. Making use of a unique feature of the Fijian Household Income and Expenditure Survey (2019/20), this paper explores the effect of respondent selection on child deprivation estimates. We compare three approaches to the selection of adult respondents commonly used in survey design: the children’s mother, the household head, and a random adult respondent in the household. We find that adults largely agree on whether children are deprived of specific items. However, in between 5 to 24% of households (depending on the indicator), adults provide different responses regarding whether children have specific items. Despite these differences, respondent selection does not substantially alter child multiple deprivation estimates or the socio-demographic characteristics of the deprived child population. The article will be of interest to those designing surveys or questionnaires to measure child poverty and children’s unmet needs.

**Supplementary Information:**

The online version contains supplementary material available at 10.1007/s12187-023-10025-1.

## Introduction

Reducing child poverty is arguably one of the most pressing issues worldwide. UNICEF estimates that one billion children lack access to basic necessities such as water, sanitation or a nutritious diet. Growing up in poverty is associated with negative consequences in terms of health, wellbeing, educational attainment and long-term economic outcomes (Cantillon et al., 2017; Gregg & Machin, 2001; Main, 2019; Pillas et al., 2014). Thus, reducing child poverty is essential to improve equality in both the short and long term.

Designing effective policies to reduce child poverty requires the correct identification of the poor. Child poverty estimates also allow tracking of the evolution of child poverty over time and how its prevalence is related to changes in social and economic conditions. The growing awareness of child poverty as a global issue, alongside the recognition that children have specific needs (Chzhen et al., 2018; De Neubourg et al., 2013; Gordon & Nandy, 2012) and growing evidence on intra-household inequality in access to resources (Brown et al., 2018; Lanau & Fifita, 2020; Main & Bradshaw, 2016) led to the development of child-specific poverty measures. Such measures capture children’s access to a range of items and activities widely viewed as necessities. Examples are the UNICEF extreme child poverty measure (Gordon et al., 2003), the EU child deprivation measure (Guio et al., 2018) and child-specific variations of the Multidimensional Poverty Index (MPI) (Osei & Turkson, 2022).

The growing interest in child poverty measurement has resulted in the development of a variety of instruments and approaches. Recent advances emphasise the need to include children’s voices when measuring child poverty by collecting data directly from children (Bessell, 2021; Chzhen et al., 2018; Main, 2018). Multiple surveys collect data from children directly, often in school settings. Examples include the Programme for International Student Assessment, Global School-based Student Health Survey, Health Behaviour of School Children and the International Survey of Children's Well-Being. These have contributed to the development of indicators that capture social phenomena from children’s perspectives (Casas et al., 2022; Chzhen et al., 2018; Main, 2019).

However, pragmatic reasons mean that child poverty estimates are most often derived from wider household surveys completed by adult respondents (i.e. people aged 18 and older in many countries). This allows for a time reduction in completing questionnaires and a side-stepping of some of the ethical and practical difficulties of collecting survey data from children.^1^ Studies comparing the responses of adolescent children and adults within the same household found both groups largely agree on whether children have material items and social activities, with larger differences regarding which items constitute necessities. In Hong Kong, (Lau et al., 2019) adults were more likely to identify all deprivation items as necessities than their children while, in the UK, Main (2018) found children placed more emphasis on “fitting in” and leisure compared with adults. Studies on this topic are comparatively limited and more research is necessary to assess to what extent adult responses adequately reflect their children’s experiences.

The use of adult respondents to measure child deprivation also opens questions regarding the effect of adult respondent selection on child poverty estimates. There are three main approaches to the selection of an adult respondent: (1) the Mother/main carer of the children, (2) the Head of Household and (3) the Random Adult in the household approaches. The first approach upholds the widely held view that mothers/main carers know best about their children's needs and wellbeing. For example, the Demographic and Health Surveys and the UK 2012 Poverty and Social Exclusion Survey adopt the ‘Mother knows best’ approach. However, many Household Income and Expenditure Surveys (HIES), often ask the household head or the person responsible for the accommodation about the needs of children in their households (Guio et al., 2018; Lanau & Fifita, 2020). The third approach involves the use of randomisation methods, such as the Kish Grid or the Birthday method, to select a household respondent as a means to obtain results that are representative at both the household and the individual level without having to interview all household members, e.g., the European Social Survey (Díaz de Rada, 2021). Since household surveys are most commonly used to produce official poverty estimates, it is important to determine the effect of (adult) respondent selection on child poverty estimates.

The extent to which the method of selection of the adult respondent affects child poverty estimates has received little attention. However, differences in adult perceptions regarding children’s access to socially defined necessities have the potential to substantially alter child deprivation estimates. Taking advantage of a unique feature of the 2019/20 Fiji Household Income and Expenditure Survey, where child deprivation questions were answered by all adults in the household, this paper examines the importance of respondent selection in child deprivation estimates. Specifically, we contrast the three approaches set out above. For each respondent selection approach, we compare and assess the effect of respondent selection on adult responses regarding whether children have an item or activity (sometimes called a ‘rights-based approach’) and on ‘enforced lack’, whether children lack the item because their households cannot afford it (e.g., the EU child deprivation measure). Furthermore, this double lens also allows assessment of the extent to which differences in response reflect differences in perception as to whether children have specific items versus differences in parental preferences about what their children should have.

Researchers aim to use reliable measures so that the same outcome of interest measured at different times or from different respondents would produce similar results (Wetzel et al., 2016). Where different measures produce different results, such results should not be trusted. Reliability is an essential prerequisite for research results to be comparable. In this case, if some individuals within the household are more likely than others to report children as deprived, child deprivation data obtained from studies relying on different household respondents (mother, head or random) may not be easily comparable.

The remainder of the paper is organised as follows. The next section provides a brief overview of the literature on child deprivation with a focus on measurement. Next, we introduce the limited literature on child poverty and deprivation in Fiji. Then, we provide details on the dataset and methods used and proceed to present the results. We find that, while in some cases there are substantial differences in adult responses within households, respondent selection has a limited impact on child poverty estimates, regardless of whether a rights or enforced lack approach is used. The conclusion assesses the implications of the findings for child poverty measurement.

## Background

Fiji is a middle-income, Melanesian country and the most populated of the island nations in the South Pacific. It has a young population with nearly 35% of Fijians under 18 (FBoS, 2021c). Fiji has two dominant cultures: the i’Taukei (indigenous Fijians) and the Indo-Fijians (Meo-Sewabu, 2021). Multi-family households are common in Fiji and children tend to live with their parents into adulthood while the elderly rarely live alone. However, nuclear families are increasingly common, particularly among the working age population in urban areas and among the Indo-Fijians. According to data from the latest HIES (2019/20), 42% of households with children are formed by six people or more. In 52% of households, there are three or more adult respondents. The high proportion of multi-adult and multi-family households makes Fiji an ideal setting for the analysis of differences in response patterns among adults.

Poverty rates in Fiji have decreased during the last two decades. For 2019, the most recent data available, 24% of the population lived in poverty according to the official definition (World Bank, 2022).^2^ The Fiji Bureau of Statistics (FBoS) estimates that 94% of medium and large businesses in Fiji were negatively affected by COVID-19 (FBoS, 2021b), suggesting that economic progress may have been stalled or reversed by the pandemic. Unfortunately, existing data does not allow estimation of the effect of COVID-19 (and the corresponding decrease in tourism and remittances) on poverty levels.

Within the traditional i’Taukei context, children are considered the responsibility of the parents as well as their extended family. Maternal and paternal relatives play a role in the protection as well as education and disciplining of the children. Meo-Sewabu (2021) highlights how the role family webs play in childrearing carries the potential to protect children, as adults share care obligations, but also makes them more vulnerable, e.g., to violence. In both the i’Taukei and Indo-Fijian cultures, a girl child is expected to contribute to the well-being of her family and community, e.g., through participation in household chores. Such tasks are seen as key to preparing girls for adulthood but can also make them more vulnerable to violence in the household (Meo-Sewabu, 2021). However, adolescent boys (13 to 17) are more likely than girls their age to have experienced serious injuries (WHO, 2020). Girls’ increased household burden does not translate into reduced educational attainment: Fiji has largely achieved gender parity in education and girls on average outperform boys in educational attainment (ADB, 2015).

In line with the findings from other contexts, children in Fiji are more likely to live in poverty compared with adults (UNICEF Pacific & Fiji Ministry of Women Children and Poverty Alleviation, 2015). Child poverty is more common in rural areas and in the Northern Division. In the last decade, the Fijian Government has progressively introduced a range of social protection schemes addressed at vulnerable families. The Care and Protection Allowance introduced by the Fijian Government aims to reduce child poverty through cash transfers and food vouchers. The programme is highly targeted which limits its potential impact on poverty reduction. The most recent estimates published by UNICEF suggest that, in 2014,the programme reached only around 2% of children (about 5,000) of the 35% estimated to live in poverty at the time (UNICEF Pacific & Fiji Ministry of Women Children and Poverty Alleviation, 2015).

## Child Poverty Measurement

Previous research on child poverty in Fiji has relied on a combination of household indicators (e.g., on overcrowding, access to radio, TV) and indicators of educational attainment and child labour (UNICEF Pacific & Fiji Ministry of Women Children and Poverty Alleviation, 2015). This approach is justified in that children are affected by the standard of living of the household in which they live. Following the adoption of the consensual approach for the measurement of adult and child deprivation by the South Pacific Statistic Methods board (FBoS, 2021a), the 2019/20 HIES included, for the first time, a child-specific set of deprivation indicators.

Deprivation indices are one of the most widely used measures of multidimensional poverty. Deprivation measures the ability of individuals or households to afford a range of socially defined needs (Guio et al., 2018; Mack & Lansley, 1985). Deprivation is a concept that cannot be observed or measured directly, rather the answers on a range of deprivation indicators are aggregated to create an index that intends to capture an underlying condition: deprivation caused by poverty.

These measures often rely on the notion of ‘enforced lack’, by which individuals and/or households are only considered to be deprived of an item if they do not have the item because they cannot afford it (but not for other reasons). ‘Enforced lack’ is widely used as a mechanism to distinguish poverty from preference, e.g., in the European Union material deprivation index (Guio et al., 2012).

The child deprivation literature has contested the use of ‘enforced lack’ to measure child deprivation on various grounds. Rights-based approaches such as UNICEF’s Multiple Overlapping Deprivation Analysis (MODA) (Chzhen et al., 2016; De Neubourg et al., 2013) or the ‘Bristol Method’ (Gordon & Nandy, 2012), emphasise that, for example, education and a decent standard of living are essential components of children’s rights and as such all children are entitled to them regardless of (parental) preference (Chzhen et al., 2016; De Neubourg et al., 2013). Indeed, in cases where adults are responding to the questionnaires on their children’s behalf, the consideration as to why children lack an item (e.g., do not want it) may reflect parental preference rather than that of the child. On this basis, the MODA framework proposes that children should be considered as deprived when they lack an item for whatever reason. Here, we assess the impact of respondent selection on deprivation measures using both the enforced lack and rights approaches.

Survey instruments always carry a degree of error. Differences in responses between subjects (or between the same subjects over time) have been associated with differences in individual characteristics (e.g., agreeability), recall error and recording errors, among other factors (Wetzel et al., 2016). Thus, differences in responses within households are expected even in a hypothetical situation where all respondents have full information and employ the same framework of reference. To address these issues and assess the consistency of responses across respondents or interrater reliability, we use a range of methods. These are discussed in more detail in the methodology section that follows.

## Methods

The article aims to assess whether and how respondent selection affects child deprivation estimates. It does so by answering two specific questions: To what extent do adults in the household agree with regard to child deprivation/whether children have a specific item? How do any differences affect deprivation estimates? For the latter, we examine both deprivation estimates using an aggregate social and material deprivation index and the effect on individual deprivation items.

### Data

To assess the degree of variation in responses about child deprivation between adults, we analyse unique Fijian data that collected responses on child deprivation from all respondent adults in the household (see Table 4 in Appendix 1 for complete list of items). Adults (aged 18 +) were asked to report about the situation of all children (under 18) in the household as a whole (which may include their own as well as other children), as opposed to collecting responses for individual children. This approach represents a limitation as well as an advantage. The limitation is that it is not possible to analyse intra-household differences in child deprivation. The advantage is that all adults in the household refer to the same group of children and thus could provide the same answers.

To assess whether the selection of the household respondent alters child deprivation estimates, we compare three approaches commonly used in research for the development of child deprivation indices: mother knows best, household head/respondent and random adult household respondent. To be able to perform this comparison, the analyses are limited to households where a mother can be identified (94% of households with children).

The Fiji HIES (2019/20) questionnaire collects information about each individual relation to the person regarded (by the members of the household) as its head distinguishing between head, spouse, son/daughter, son/daughter in law, grandchild, parents, parent-in-law, brother/sister, adopted/foster child, other relatives and not related. As a result, it is not always possible to identify parenthood relationships for non-head household members in multi-family households or households with extended co-residence. In slightly over 10% of households with children, multiple mothers can be identified within the same household. In those cases, when using the children’s mother approach, children are considered to be deprived/lack an item if one household mother labels them as deprived/not having the item. Only in 1 to2% of the cases is there a disagreement between different mothers in the household as to whether the children are deprived/lack an item.

In the deprivation module, adults (18 +) are presented with three questions for any given item: whether the item is a necessity, whether children in the household have the item and if not, why not? The unit of analysis is all households with children (N = 3,370). Adults are encouraged to think about all children in the household when replying to the child deprivation questions.

Table 1 describes key characteristics of the sample used in the analyses. The majority of households in the sample have two or more children, 39% have one child, 2% have six or more children. In 95% of households, there are two or more respondents per child deprivation item. This allows examination of variations in adult responses. In 44% of households, there are two respondents. In more than 50% of households, there are three or more respondents and, in 14% of households, five or more. This diversity of household compositions allows the examination of variations in respondent selection under different conditions. The vast majority of households (84%) are headed by a man.

**Table 1:** Sample characteristics. Households with children

		%
Household head	Female	16
	Male	84
Number of children	1	39
	2	30
	3	17
	4	9
	5	3
	6 +	2
Number of respondents	1	4
	2	44
	3	22
	4	16
	5 +	14

Source: Authors’ own using Fiji HIES (2019/20)

The Fiji HIES collects information on nine child deprivation items. In analyses published by (FBoS (2021a), eight of these items were considered appropriate indicators of deprivation in Fiji. These were three meals a day, a meal with protein, new properly fitting shoes, some new clothes (not second-hand), participating in school trips and events that cost money, celebrations on special occasions, beds and bedding for every child and a place to do homework. These items have been used to build the child deprivation index. One further item, having a bicycle, was included in the questionnaire but is not widely perceived as a necessity for children in Fiji. Only 14% of adults consider a bicycle to be a necessity, while 20% consider it to be neither necessary nor desirable, which may reflect under-developed infrastructure for bicycle use. As a result, this item is excluded from the deprivation index, as it lacks face validity in Fiji, but it is included in the initial analyses as it provides additional information on response patterns.

To be able to assess the potential impact of respondent selection on deprivation estimates, two summative indices are calculated that range between 0 and 8. The ‘enforced lack’ index considers children to be deprived of an item when they lack the item due to affordability reasons. The ‘rights based’ index counts, as deprived, children who lack an item for any reason. As results can be influenced by the threshold value used, we test the robustness of the findings by reporting the results for two thresholds that classify as ‘deprived’ those children lacking 3 + or 5 + items. Analyses were also calculated for a 2 + threshold but, given the consistency of results, these are not presented here for brevity.

### Assessing Consistency of Responses

Multiple respondents may interpret a phenomenon of interest differently. In this case, mothers, household heads and selected random adult respondents may provide conflicting information as to whether children in their households are deprived. For each item, we report the proportion of households where all three respondents provide the same reply. We also use Bland–Altman plots to identify any items that might be problematic. To assess the degree of similarity between answers provided by different respondents, we use a range of measures designed to assess interrater reliability, the extent to which different people evaluate a situation similarly (McHugh, 2012). We estimate three measures widely used in the literature: percent agreement, Fleiss kappa and Gwet’s AC_1_. Percent agreement represents the proportion of cases in which respondents provide the same answer (e.g., has/has not). With three or more respondents, the statistic produces the average agreement among pairs of respondents. This provides an intuitive answer as to the degree of similarity across responses. Kappa and Gwet’s AC_1_ are agreement coefficients, providing a reference point for assessing agreement with a standardized value that can be compared across measures or studies: 1 represents perfect agreement and 0 the amount of agreement that can be expected by chance. Generally, values above 0.6 are considered substantial agreement and, above 0.8, as almost perfect agreement (McHugh, 2012). Kappa is arguably the most widely used agreement statistic but tends to perform poorly in cases where the probability of an event is either very high or very low. This is an issue for items that capture severe forms of deprivation and that only a small proportion of children lack, such as three meals a day. Gwet’s AC_1_ corrects for that limitation (Wongpakaran et al., 2013).

## Findings

This section reports on the effect of respondent selection on child deprivation estimates. The presentation of findings is structured in three subsections each addressing one sub-question: (1) How common is disagreement in adult perceptions of child deprivation? (2) Which factors predict disagreement? and (3) How significant is it for child deprivation estimates? The first two sub-sections focus on agreement at the item level, while the third section examines the effect on child deprivation estimates using aggregate indices.

### Are we All Agreed? Adults’ Perception of Child Deprivation

Table 2 shows the proportion of households where there is a mismatch in responses, that is, where not all adults in the household have provided the same response regarding a given item. The first column reports differences observed when using the enforced lack criteria and the second when using a rights approach, i.e., where a lack for any reason is considered as deprivation. As a general rule disagreement is larger for items where deprivation is more common, although the relationship is not linear (see Table 5 in Appendix 2).

**Table 2:** Households where a mismatch between adult responses is recorded (% of households)

	Enforced lack		Rights based	
Three meals	5	[4–6]	8	[6–9]
Beds and bedding	11	[10–13]	17	[15–18]
Homework	12	[10–13]	19	[17–21]
Meat	14	[12–17]	17	[15–19]
Celebrations	14	[13–16]	21	[19–23]
School trips	15	[13–17]	26	[24–28]
Shoes	16	[15–18]	24	[22–26]
Clothes	18	[17–20]	23	[21–25]
Bicycle*	28	[26–30]	12	[10–13]

* Bicycle is not widely considered as a necessity and thus it is not included in the deprivation indices. Confidence intervals calculated using complex samples. Mismatch or disagreement identifies the proportion of households where adults provide different responses regarding whether children in the household lack/are deprived of an item.

Source: Authors’ own using Fiji HIES (2019/20)

In the majority of households, adults agree whether children are deprived of an item. The proportion of households where there is a difference in adult responses varies by item ranging from 5% for three meals a day to 28% for bicycle in the case of ‘enforced lack’, and between 8 and 26% in the case of all lacks (Table 2). When ‘bicycle’ (an item that is not widely viewed as a necessity) is excluded, in over 80% of households, adults provide the same response across items.

For three deprivation items: shoes, clothes and school trips, in around one in four households there are disagreements as to whether children have these items. Thus, in a minority of households, disagreements are observed. These disagreements amongst respondents in a minority of households may signify different knowledge of the situation of children in the household but also that adults may be thinking of different children when responding to the questionnaire. For instance, some adults may consider children have some new clothes if the eldest received some new clothes that can then be passed down to younger siblings, while others may weight all individual children in their response. We find no gender differences in the probability of identifying children as having an item. Respondent’s age does show some association with the probability of identifying children as deprived. Specifically, the 31 to 40 age group is the most likely to respond positively when asked whether children have an item, while older respondents (50 +) are the least likely to say so. Thus, the age group most likely to be parents are also the most likely to identify children as not being deprived. The largest differences between age groups are recorded for clothes, shoes and school trips and the smallest ones for bicycle. In the absence of an objective measure, it is not possible to ascertain whose answers are most likely to reflect children’s ‘true’ deprivation status. In any case, differences are generally small and, as discussed in the next section, do not appear to significantly alter child deprivation estimates.

The proportion of households where there is a mismatch or inconsistency in adults’ responses is generally smaller when using the enforced lack approach than with the rights approach. This result is counterintuitive, as one would expect adults to differ less on whether children have an item (observable) than on their preferences or perception as to why a particular item is lacking. Instead, the opposite is observed. The explanation lays with the increase in measurement reliability (e.g., the reduction in the amount of random measurement error) when respondents are allowed to distinguish choice from constraint. It is known, from research in multiple countries, that the enforced lack method produces more reliable results than the ‘any lack’ rights approach (Guio et al., 2012). The reduction in random measurement error in the enforced lack approach results in less interrater variability within households.

As an aside, the high prevalence of disagreement regarding whether children have a bicycle is interesting in that a bicycle is a large, visible object. One might expect less disagreement with such an object compared with something abstract (celebrations) or that does not happen in the house (e.g., school trips) or smaller items (e.g., shoes). It is likely that bicycles are shared within the household, making it harder to determine ‘ownership’ The next section examines the factors associated with inconsistent responses.

### Item By Item Variation in Adult Responses

While deprivation is measured through aggregated indices, analysts may be interested in the proportion of children who lack a specific item or sets of items. This can provide additional information regarding which forms of deprivation are more common in the population and help identify potential policy responses.

**Fig. 1:**
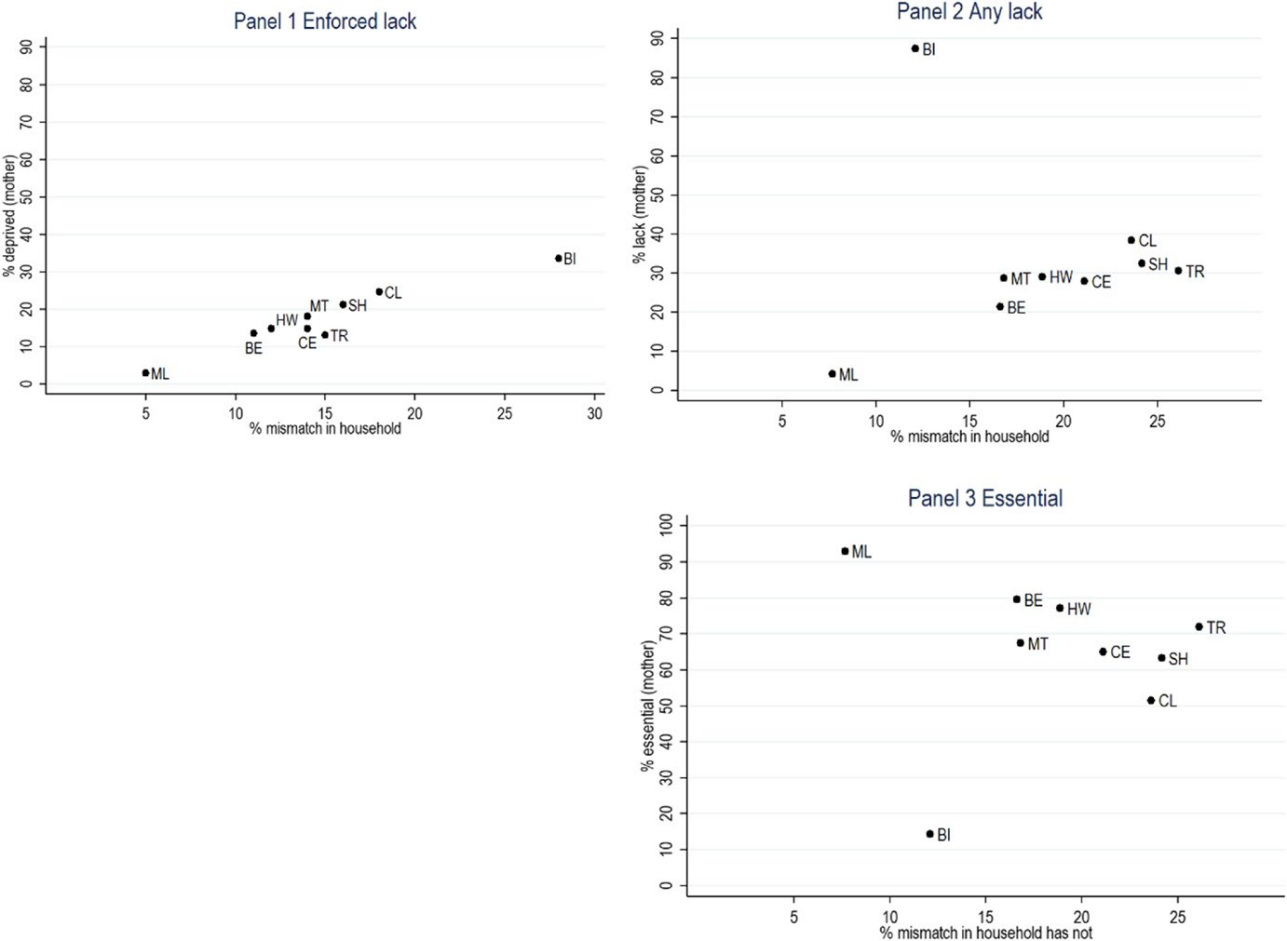
Association between within household mismatch and enforced lack deprivation, any lack deprivation and the proportion of respondents who view the item as essential. Labels: ‘ML’ Three meals a day, ‘MT’ meat or protein, ‘SH’ shoes, ‘CL’ clothes, ‘TR’ school trips, ‘BI’ Bicycle, ‘BE’ Beds and bedding, ‘HW’ Homework space, ‘CE’ Celebrations. Source: Authors’ own using Fiji HIES (2019/20)

Figure 1 shows, for each child deprivation item, the proportion of households where differences in responses are observed. This is represented on the horizontal axis. The mismatch or disagreement indicator corresponds to column 2 in Table 2 and thus reflects mismatch under the rights approach. The vertical axis shows the proportion of children who are deprived according to the ‘enforced lack’ approach (panel 1), the rights-based approach (panel 2) and the proportion of the population who consider the item a necessity (panel 3). Since estimates are similar across respondents, we report here mothers’ responses, i.e., the assumed ‘gold standard’.

The first thing that stands out is that ‘bicycle’ is an outlier in all three figures. A large number of children lack the item and very few mothers consider it as essential. The item was excluded from the official child deprivation index as it was considered a poor indicator of child deprivation in Fiji. The figures indicate that patterns of association between mismatch or differences within the household and deprivation levels are also different from those of other items.

Eight other items were included in the index. If we examine the vertical axis, deprivation rates are lowest for three meals a day (3–4% of children depending on the indicator and highest for some new clothes, 25% with the ‘enforced lack’ indicator and 38% with the rights approach). By definition, deprivation rates are higher when using the ‘rights based’ measure compared to the ‘enforced lack’ measure, although the size of the gap varies widely by indicator.

The figures suggest that differences in perception of child deprivation are larger for items where there is more disagreement amongst adults about whether they are essential for children (bottom scatterplot). The top two scatterplots show a weak correlation between the number of children lacking an item (% deprived) and the amount of disagreement amongst adults in the household (% mismatch).

**Fig. 2:**
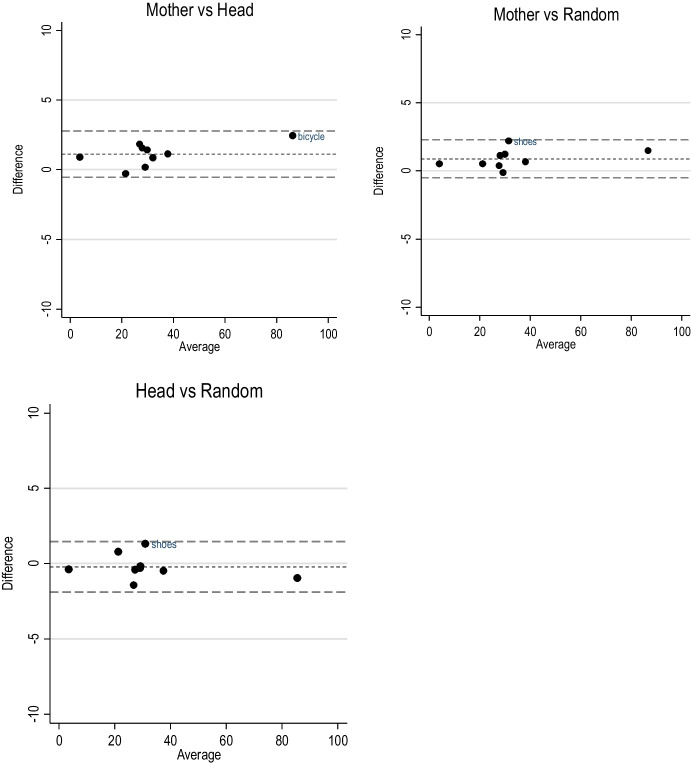
Bland–Altman plots for repondent pairs. Source: Authors’ own using HIES (2019/20)

To further explore and illustrate differences between respondents for individual items, we present Bland–Altman plots (Bland & Altman, 1986) (Fig. 2). Bland–Altman plots reflect whether differences between groups (represented in the vertical axis) can be considered as significant or, on the contrary, the variation falls within the expected random variation, represented by the two horizontal dashed lines. The method only allows for comparison of differences between two respondents or groups at a time. Thus, we have conducted the analysis for each pair of respondents: mother and household head, mother and random and head and random. The horizontal axis reflects the arithmetic mean between the two groups, here the average of how often children are identified to lack the item. The plots allow identification of any items that may be particularly problematic. Overall, the figures suggest no substantial differences between respondents. Two items, ‘bicycle’, when comparing mother and head, and ‘shoes’ (mother vs random and mother vs head), appear as borderline but remain within normal bounds. Respondent selection does not seem to affect conclusions regarding item deprivation.

So far, analyses have focused on the item-level predictors of differences in adult response. We finalise our analysis by exploring the association between household characteristics and differences in adults’ responses using logistic regression models. The dependent variable takes the value 1 when discrepancies in adult responses are observed for a specific item and zero when all adults provide the same response. A separate regression is run for each item. As independent variables, we include number of respondents in the household, number of generations, number of children and income poverty status (as defined by the national poverty measure). The results of the models for each item are reported in Table 6 in Appendix 3.

Perhaps unsurprisingly, the strongest predictor of disagreement is the number of respondents. Each additional respondent in the household is significantly associated with an increased probability of having mismatched responses in the household. Household poverty appears to be significant for some items but not others. Adults in poor households are more likely to disagree about whether children have three meals a day and school items. By contrast, poor households show less disagreement with regards to ‘bicycle’ than other households. For the remaining items, there are no differences between poor and non-poor households. Number of children and number of generations living in the household do not appear to make a difference to the probability of observing a disagreement. The following section examines to what extent the observed differences affect child deprivation estimates.

### Respondent Selection and Deprivation Measures: Does It Matter Who You Ask?

Deprivation is usually measured through summative indices aimed at capturing multidimensional poverty. Children who are deprived of a number of items are categorised as multiply deprived. It is important to know if the respondent selection method results in different poverty estimates when calculating deprivation indices, whether the same households are identified as poor and whether the same conclusions are reached regarding the characteristics of the poor population.

Table 3 reports child deprivation rates with two thresholds (lacking 3 + and 5 + items). As expected, deprivation estimates decrease as the threshold becomes more severe. As would be expected, estimates produced using the rights approach are substantially higher than those obtained using ‘enforced lack’, in some cases almost double.

**Table 3:** Child deprivation rates by indicator, threshold and selected respondent

		3 +		5 +	
Enforced lack	Mother	20	[18–23]	10	[8–12]
	Head	18	[16–20]	8	[6–9]
	Random	18	[17–21]	9	[7–11]
Rights approach	Mother	37	[34–40]	17	[15–20]
	Head	36	[33–39]	16	[14–18]
	Random	36	[33–39]	16	[14–18]

Confidence intervals calculated using complex samples.

Source: Authors’ own using HIES (2019/20)

The method used to select the household respondent appears to have no significant effect on estimated deprivation prevalence rates. Mothers are slightly more likely to identify children as deprived but the difference is small and not statistically significant.

We next tested whether the different approaches to respondent selection identify the same children/households as deprived. The first line in Table 4 shows the proportion of cases that are classified consistently by all three methods of selecting respondents. We found that, in between 89 and 94% of cases, depending on the indicator, we obtained consistent classifications across all three household respondents. Higher levels of agreement were found for enforced lack compared with the rights-based method and with more severe thresholds. These results were confirmed by the interrater agreement statistics (Kappa and AC1). Percent agreement, Kappa and Gwet’s AC_1_ all indicated a very high level of agreement. Lower values for Kappa for the more severe 5 + threshold were expected, as Kappa is known to under-estimate agreement when a high proportion of responses fall in one of the categories.

**Table 4:** Inter-rater reliability amongst household respondents of child deprivations

	Enforced lack		Rights approach	
Threshold	3 +	5 +	3 +	5 +
All respondents agree	89%	94%	84%	89%
Percent Agreement	93%	96%	89%	92%
Scott/Fleiss’ Kappa	0,76	0,72	0,77	0,71
Gwet’s AC_1_	0,9	0,95	0,8	0,89

Source: Authors’ own using HIES (2019/20)

Thus, while deprivation estimates are very similar, independent of the method used to choose the selected respondent, between 11 and 16% of cases, depending on the indicator, are classified differently. Agreement is highest in households with 2 respondents but remains high with 4 or more respondents (Table 7 in Appendix 4). This suggests our results are robust to variations in the number of respondents per household across contexts/surveys.

Finally, we considered whether respondent selection altered the groups identified as poor in terms of characteristics commonly associated with poverty. Estimates are reported for ‘all lacks’ (the rights approach) as it is the category where differences are more substantial. The results, shown in Table 8 in Appendix 5, are remarkably similar in terms of gender of the household head, their education and labour market status, as well as household characteristics in terms of food poverty, urban/rural location and number of children.

Overall, the respondent selection method appears to have almost no effect on child deprivation estimates. This is good news for researchers, policymakers, survey designers and others interested in producing child poverty estimates from household survey data.

## Conclusion

Child deprivation indicators often rely on information reported by a selected adult regarding children’s living standards. Surveys use different criteria to choose the selected adult respondent to complete household and child related information. Adult respondent selection would affect child deprivation estimates if respondents with different characteristics or relation to children in the household are likely to provide systematically different responses. Using unique data collected in Fiji in 2019/20, this paper examines empirically whether and how respondent selection affects child deprivation estimates.

The findings are clear and reassuring: adult respondent selection does not significantly alter child deprivation estimates, irrespective of the method and thresholds used to identify multiply deprived children. It does not matter if the respondent is the children’s mother, the household head or a random household respondent – the same conclusions about child deprivation will be reached. While deprivation estimates are somewhat higher when mothers’ responses are used, the differences are small, in the range of 1% to 2% and not statistically significant. These results provide some confidence regarding the ability to compare child deprivation estimates from surveys with different approaches to respondent selection. Child deprivation estimates are robust to the respondent selection method used.

The Fijian context is interesting as the diversity of household living arrangements in the sample allows for the comparison of respondent selection effects in households with different compositions. We observe that disagreement within households increases with the number of respondents (although not with the number of children). Thus, it is to be expected that differences in child deprivation estimates would be even smaller in countries where one and two adult households are the norm, compared with Fiji where the majority of children live in households with three or more adults. That said, in the large majority of households in our sample, adults agree as to whether children are deprived of specific items.

Differences of opinion are rare for items that are largely perceived as necessities and where deprivation is low but increases to 24%-26% of households for the items where deprivation is more common. Researchers aiming at maximising agreement should choose items that are widely perceived as necessities. Disagreement regarding whether children have an item is lowest for items that are owned by most or very few children and higher for those items where there is more variation. However, it is worth underlining that the results from the interrater reliability statistics (Kappa and Gwet’s AC_1_) and the Bland–Altman plots show that these differences may be largely a result of random error in the survey as they do not exceed the expected levels of variation in a household survey of the size of the Fiji HIES (2019/20). Qualitative research might help to understand the factors behind the relatively small differences in respondents’ replies. The ‘enforced lack’ method for measuring deprivation, which separated choice from constraint as children are only identified as deprived due to inadequate household income/resources, produces more robust results and higher levels of respondent agreement than the simple lack method (‘rights based’ approach).

The conclusion from this research is that the method used to select the adult respondent in a household survey has little effect on estimates of child deprivation. While adults sometimes disagree regarding whether children have access to specific items, such differences do not alter child deprivation estimates. The estimated child multiple deprivation rate and the socio-demographic characteristics of the deprived child population are consistent irrespective of the method used for household respondent selection.

### Electronic supplementary material

Below is the link to the electronic supplementary material.

Supplementary file1 (GPH 8 KB)

Supplementary file2 (GPH 7 KB)

Supplementary file3 (GPH 7 KB)

## Data Availability

The data is owned by the Fiji Bureau of Statistics (FBoS). We cannot make any of the data available as it is owned by FBoS.

## Appendices

Tables

**Table 5:** Consensual child deprivation questions in the 2019/20 Fiji HIES. Instructions: Each item has 2 questions, for each item, ask the respondent if he/she believes the item is something everyone should possess, the second question asks whether the respondent possess the item and the reasons for not possessing (Respondents all >  = 18)

Child Items	Is (item) 1 = Essential 2 = Desirable but not essential 3 = Neither 4 = Don’t Know	Do you have (item)? 1 = Have it 2 = Don’t have, can’t afford 3 = Don’t have, don’t want 4 = Don’t have, for another reason 5 = Don’t know
Q1. Three meals a day		
Q2. One meal with meat, chicken or fish or vegetarian equivalent daily		
Q3. New, properly fitting shoes		
Q4. Some new, not second-hand clothes		
Q5. Participate in school trips and school events that cost money		
Q6. Bicycle		
Q7. Enough beds and bedding for every child in the household		
Q8. A suitable place at home to study or do homework		
Q9. Celebrations on special occasions such as birthdays, Christmas or religious festivals		

Source: FBoS, 2021a, p. 89

^5^,

**Table 6:** Households where a mismatch between adult responses is recorded and deprivation rates by item (% of households)

	Disagreement (%)				Deprivation (% according to mother)			
	Enforced lack		Rights based		Enforced lack		Rights based	
Three meals	5	[4–6]	8	[6–9]	3	[2–4]	4	[3–5]
Beds and bedding	11	[10–13]	17	[15–18]	14	[12–16]	21	[19–24]
Celebrations	14	[13–16]	21	[19–23]	15	[13–17]	28	[25–31]
Meat	14	[12–17]	17	[15–19]	18	[16–21]	29	[26–32]
Homework	12	[10–13]	19	[17–21]	15	[13–17]	29	[27–32]
School trips	15	[13–17]	26	[24–28]	13	[11–15]	31	[28–33]
Shoes	16	[15–18]	24	[22–26]	21	[19–24]	32	[30–35]
Clothes	18	[17–20]	23	[21–25]	25	[22–27]	38	[35–41]
Bicycle*	28	[26–30]	12	[10–13]	34	[31–36]	87	[85–89]

Source: Author’s own using HIES 2019/20. Note: Disagreement identifies the proportion of households where adults provide different responses regarding whether children in the household lack/are deprived of an item

6,

**Table 7:** Predictors of differences in respondents' perception of child deprivations

Logistic regression. Dependent Variable: Differences in adults respondents by item. Households with children and mother									
	Deprivation items								
	Three meals a day	Meat or Protein	Shoes	Clothes	School trips	Bicycle	Beds and bedding	Homework	Celebrations
Number of generations (ref 1)									
2 generations	-0.43	0.26	0.13	-0.29	0.23	0.3	0.07	-0.03	-0.39
3 + generations	-0.34	0.51	0.19	-0.19	0.31	0.44	0.21	0.01	-0.12
Number pf children (ref 1)									
2 children	0.06	-0.03	0.12	0.14	0.05	0.1	0.1	0.01	0.08
3 children	0.11	-0.13	-0.1	-0.02	0.11	-0.29	0	0.11	0
4 + children	-0.22	0.09	0.01	0.14	-0.05	-0.15	0.05	-0.09	0.08
Poor	0.61***	-0.07	0.11	0.02	0.24*	-0.66***	0.16	-0.12	0.12
Number of respondents (ref 2)									
1	0.17	0.23	-0.38	0.07	0.12	-1.92**	0.39	-0.03	-0.27
3	0.73***	0.54***	0.79***	0.81***	0.93***	0.39*	0.67***	0.76***	0.57***
4	1.08***	0.88***	1.20***	1.07***	1.12***	0.74***	0.79***	0.97***	0.82***
5 +	1.22***	1.14***	1.44***	1.45***	1.29***	1.22***	1.28***	1.28***	0.93***
_constant	-2.91***	-2.41***	-1.99***	-1.64***	-2.02***	-2.57***	-2.35***	-1.98***	-1.52***
N	3369	3365	3365	3358	3351	3334	3357	3347	3323
PseudoR	0.052	0.042	0.062	0.057	0.056	0.051	0.042	0.041	0.04

Source: Author's own using HIES 2019/20

^7^,

**Table 8:** Agreement statistics by number of respondents (3 + threshold)

		Number of respondents				
		1	2	3	4 +	All
Enforced lack	All respondents agree	1	0.91	0.87	0.85	0.89
	Percent Agreement	1	0.94	0.92	0.90	0.93
	Scott/Fleiss' Kappa	1	0.81	0.74	0.71	0.76
	Gwet's AC	1	0.92	0.88	0.86	0.9
All lacks	All respondents agree	1	0.87	0.83	0.78	0.84
	Percent Agreement	1	0.91	0.89	0.85	0.89
	Scott/Fleiss' Kappa	1	0.87	0.76	0.69	0.77
	Gwet's AC	1	0.84	0.80	0.72	0.8

Source: Author’s own using HIES 2019/20

^8^,

**Table 9:** Characteristics of households with deprived children according to selected respondent (3 + threshold, all lacks)

		Respondent		
		Mother	Head	Random
Household head	Female	18	18	17
	Male	82	82	83
	Secondary education or more	62	62	62
	Below secondary education	38	38	38
	Not working	20	19	18
	Wage/salary earner	45	45	48
	Employer	1	1	0
	Self-employed	21	22	21
	Family/community work	1	1	1
	Subsistence	12	12	12
Food poverty	Not poor	93	93	93
	Poor	7	7	7
Location	Rural	48	48	48
	Urban	52	52	52
N children	1	34	34	35
	2	31	30	30
	3	19	19	19
	4	10	9	10
	5	4	5	5
	6 +	3	3	3

Source: Author’s own using HIES 2019/20

^9^

## Footnotes

1 These include cost reduction by having one single respondent, the need to adapt questions to specific age groups and the requirement of specific legal authorisations in some countries.

2 The original World Bank Basic Needs poverty rate of 29% was revised down to 24% after the correction of a coding error.

## Declarations

### Data

The data is owned by the Fiji Bureau of Statistics (FBoS).
